# Supplementary material for: The subcortical correlates of autistic traits in school-age children: a population-based neuroimaging study
Source: Mol Autism. 2023 Feb 11;14:6. doi: 10.1186/s13229-023-00538-5 (PMC9921646; doi:10.1186/s13229-023-00538-5)
Supplement: Supplementary file 1 — Additional file 1 Supplementary Tables. [file 13229_2023_538_MOESM1_ESM.docx]

**Supplementary Tables**

|  | **ROI** | **B** | **SE** | **p-value** | **holm p-value** |
| --- | --- | --- | --- | --- | --- |
| Model 1 | thalamus | -7.87 | 3.82 | 0.039 | 0.118 |
|  | caudate | -7.60 | 3.12 | 0.015 | 0.059 |
|  | putamen | -9.38 | 3.67 | 0.011 | 0.053 |
|  | pallidum | -3.31 | 1.14 | 0.004 | 0.023 |
|  | amygdala | -1.14 | 1.09 | 0.294 | 0.294 |
|  | hippocampus | -4.42 | 2.29 | 0.053 | 0.118 |
|  | NAcc | -1.68 | 0.54 | 0.002 | 0.013 |
| Model 2 | thalamus | -3.77 | 3.85 | 0.327 | 0.674 |
|  | caudate | -5.74 | 3.16 | 0.069 | 0.336 |
|  | putamen | -6.78 | 3.70 | 0.067 | 0.336 |
|  | pallidum | -2.63 | 1.16 | 0.023 | 0.139 |
|  | amygdala | -0.51 | 1.10 | 0.644 | 0.674 |
|  | hippocampus | -2.81 | 2.31 | 0.225 | 0.674 |
|  | NAcc | -1.51 | 0.55 | 0.006 | 0.039 |
| Model 3 | thalamus | 0.53 | 4.33 | 0.903 | 1.000 |
|  | caudate | -4.88 | 3.55 | 0.169 | 1.000 |
|  | putamen | -5.20 | 4.17 | 0.213 | 1.000 |
|  | pallidum | -1.93 | 1.30 | 0.139 | 0.975 |
|  | amygdala | 0.95 | 1.24 | 0.441 | 1.000 |
|  | hippocampus | 1.40 | 2.60 | 0.590 | 1.000 |
|  | NAcc | -0.79 | 0.61 | 0.199 | 1.000 |

*Table S1: Results from regression modelling of the association between the SRS and subcortical ROIs. Corrected p-values were generated using holm correction for multiple testing. NAcc: nucleus accumbens. Model 1 was adjusted for child age, sex, ethnicity, family income, and ABCD recruitment site. Model 2 was adjusted for the covariates included in model 1 with the addition of cognition score. Model 3 was adjusted for the covariates included in model 2 with the addition of externalising symptoms and internalising symptoms*

|  |  |  |  |  |  |
| --- | --- | --- | --- | --- | --- |
|  | **ROI** | **B** | **SE** | **p-value** | **holm p-value** |
| Model 1 | thalamus | -0.98 | 2.69 | 0.72 | 1.00 |
|  | caudate | -3.35 | 2.63 | 0.20 | 0.81 |
|  | putamen | -4.95 | 3.23 | 0.13 | 0.63 |
|  | pallidum | -1.86 | 0.99 | 0.06 | 0.37 |
|  | amygdala | 0.27 | 0.94 | 0.78 | 1.00 |
|  | hippocampus | -1.28 | 1.92 | 0.51 | 1.00 |
|  | NAcc | -1.00 | 0.47 | 0.03 | 0.22 |
| Model 2 | thalamus | 0.18 | 2.73 | 0.95 | 1.00 |
|  | caudate | -3.29 | 2.67 | 0.22 | 0.95 |
|  | putamen | -4.25 | 3.27 | 0.19 | 0.95 |
|  | pallidum | -1.80 | 1.01 | 0.07 | 0.42 |
|  | amygdala | 0.30 | 0.95 | 0.75 | 1.00 |
|  | hippocampus | -1.01 | 1.95 | 0.61 | 1.00 |
|  | NAcc | -1.12 | 0.47 | 0.02 | 0.14 |
| Model 3 | thalamus | 2.35 | 3.07 | 0.44 | 1.00 |
|  | caudate | -3.75 | 3.01 | 0.21 | 1.00 |
|  | putamen | -4.03 | 3.68 | 0.27 | 1.00 |
|  | pallidum | -1.54 | 1.13 | 0.17 | 1.00 |
|  | amygdala | 1.33 | 1.07 | 0.21 | 1.00 |
|  | hippocampus | 2.23 | 2.19 | 0.31 | 1.00 |
|  | NAcc | -0.61 | 0.53 | 0.26 | 1.00 |
|  |  |  |  |  |  |
|  |  |  |  |  |  |

*Table S2: Results from regression modelling of the association between the SRS and subcortical ROIs with the inclusion of ICV. Corrected p-values were generated using holm correction for multiple testing. NAcc: nucleus accumbens. Model 1 was adjusted for child age, sex, ethnicity, family income, and ABCD recruitment site. Model 2 was adjusted for the covariates included in model 1 with the addition of cognition score. Model 3 was adjusted for the covariates included in model 2 with the addition of externalising symptoms and internalising symptoms.*

| **Model** | **ROI** | **Hemisphere** | **B** | **SE** | **p-value** | **holm p-value** |
| --- | --- | --- | --- | --- | --- | --- |
| Model 1 | thalamus | LH | -0.56 | 1.44 | 0.70 | 1.00 |
|  |  | RH | -0.42 | 1.45 | 0.77 | 1.00 |
|  | caudate | LH | -1.48 | 0.69 | 0.03 | 0.43 |
|  |  | RH | -0.38 | 0.50 | 0.44 | 1.00 |
|  | putamen | LH | -1.42 | 1.35 | 0.29 | 1.00 |
|  |  | RH | -1.94 | 1.36 | 0.15 | 1.00 |
|  | pallidum | LH | -0.39 | 0.29 | 0.17 | 1.00 |
|  |  | RH | -0.62 | 0.25 | 0.02 | 0.22 |
|  | amygdala | LH | -2.61 | 1.83 | 0.15 | 1.00 |
|  |  | RH | -2.34 | 1.56 | 0.13 | 1.00 |
|  | hippocampus | LH | -0.69 | 1.08 | 0.52 | 1.00 |
|  |  | RH | -0.59 | 1.02 | 0.56 | 1.00 |
|  | NAcc | LH | 0.40 | 0.53 | 0.44 | 1.00 |
|  |  | RH | -0.14 | 0.53 | 0.80 | 1.00 |
| Model 2 | thalamus | LH | 0.22 | 1.47 | 0.88 | 1.00 |
|  |  | RH | -0.03 | 1.47 | 0.98 | 1.00 |
|  | caudate | LH | -1.48 | 0.71 | 0.04 | 0.48 |
|  |  | RH | -0.32 | 0.50 | 0.52 | 1.00 |
|  | putamen | LH | -1.37 | 1.37 | 0.32 | 1.00 |
|  |  | RH | -1.92 | 1.38 | 0.16 | 1.00 |
|  | pallidum | LH | -0.45 | 0.29 | 0.12 | 1.00 |
|  |  | RH | -0.67 | 0.26 | 0.01 | 0.13 |
|  | amygdala | LH | -2.23 | 1.85 | 0.23 | 1.00 |
|  |  | RH | -2.02 | 1.58 | 0.20 | 1.00 |
|  | hippocampus | LH | -0.45 | 1.10 | 0.68 | 1.00 |
|  |  | RH | -0.56 | 1.03 | 0.59 | 1.00 |
|  | NAcc | LH | 0.47 | 0.53 | 0.38 | 1.00 |
|  |  | RH | -0.17 | 0.54 | 0.76 | 1.00 |
| Model 3 | thalamus | LH | 1.12 | 1.65 | 0.50 | 1.00 |
|  |  | RH | 1.23 | 1.65 | 0.46 | 1.00 |
|  | caudate | LH | -1.28 | 0.80 | 0.11 | 1.00 |
|  |  | RH | -0.26 | 0.57 | 0.64 | 1.00 |
|  | putamen | LH | -1.34 | 1.54 | 0.38 | 1.00 |
|  |  | RH | -2.41 | 1.56 | 0.12 | 1.00 |
|  | pallidum | LH | -0.31 | 0.33 | 0.34 | 1.00 |
|  |  | RH | -0.29 | 0.29 | 0.31 | 1.00 |
|  | amygdala | LH | -2.25 | 2.08 | 0.28 | 1.00 |
|  |  | RH | -1.77 | 1.78 | 0.32 | 1.00 |
|  | hippocampus | LH | 1.21 | 1.23 | 0.32 | 1.00 |
|  |  | RH | 1.02 | 1.16 | 0.38 | 1.00 |
|  | NAcc | LH | 0.96 | 0.60 | 0.11 | 1.00 |
|  |  | RH | 0.37 | 0.61 | 0.55 | 1.00 |

*Table S3:* *Results from regression modelling of the association between the SRS and subcortical ROIs by hemisphere with the inclusion of ICV. Corrected p-values were generated using holm correction for multiple testing. NAcc: nucleus accumbens. Model 1 was adjusted for child age, sex, ethnicity, family income, and ABCD recruitment site. Model 2 was adjusted for the covariates included in model 1 with the addition of cognition score. Model 3 was adjusted for the covariates included in model 2 with the addition of externalising symptoms and internalising symptoms.*

|  | **ROI** | **B** | **SE** | **p-value** | **holm p-value** |
| --- | --- | --- | --- | --- | --- |
| **Internalising symptoms only** | thalamus | 1.13 | 3.01 | 0.71 | 1 |
|  | caudate | -3.34 | 2.95 | 0.26 | 1 |
|  | putamen | -3.17 | 3.62 | 0.38 | 1 |
|  | pallidum | -1.53 | 1.12 | 0.17 | 1 |
|  | amygdala | 1.22 | 1.05 | 0.25 | 1 |
|  | hippocampus | 1.13 | 2.16 | 0.6 | 1 |
|  | NAcc | -0.67 | 0.52 | 0.2 | 1 |
| **Externalising symptoms only** | thalamus | 2.69 | 2.95 | 0.36 | 1 |
|  | caudate | -4.02 | 2.89 | 0.16 | 0.78 |
|  | putamen | -5.28 | 3.54 | 0.14 | 0.78 |
|  | pallidum | -1.71 | 1.09 | 0.12 | 0.78 |
|  | amygdala | 0.89 | 1.03 | 0.39 | 1 |
|  | hippocampus | 1.8 | 2.11 | 0.39 | 1 |
|  | NAcc | -0.82 | 0.51 | 0.11 | 0.78 |
| **ADHD symptoms only** | thalamus | 0.25 | 3.00 | 0.93 | 1.00 |
|  | caudate | -3.24 | 2.94 | 0.27 | 1.00 |
|  | putamen | -4.86 | 3.61 | 0.18 | 0.95 |
|  | pallidum | -1.57 | 1.11 | 0.16 | 0.95 |
|  | amygdala | 0.83 | 1.04 | 0.43 | 1.00 |
|  | hippocampus | 0.99 | 2.15 | 0.65 | 1.00 |
|  | NAcc | -0.89 | 0.52 | 0.09 | 0.61 |

*Table S4: Results from regression modelling of the association between the SRS and subcortical ROIs examining internalising, externalising, and ADHD symptoms separately. Corrected p-values were generated using holm correction for multiple testing. NAcc: nucleus accumbens. Models were adjusted for child age, sex, ethnicity, family income, ABCD recruitment site, cognition score, and ICV.*

|  |  | **ROI** | **B** | **SE** | **p-value** | **holm p-value** |
| --- | --- | --- | --- | --- | --- | --- |
| Female only | Model 1 | thalamus | -7.40 | 4.22 | 0.08 | 0.50 |
|  |  | caudate | -0.18 | 4.12 | 0.96 | 1.00 |
|  |  | putamen | -6.43 | 5.08 | 0.21 | 1.00 |
|  |  | pallidum | -1.13 | 1.56 | 0.47 | 1.00 |
|  |  | amygdala | 0.99 | 1.43 | 0.49 | 1.00 |
|  |  | hippocampus | -0.16 | 3.06 | 0.96 | 1.00 |
|  |  | NAcc | -1.37 | 0.76 | 0.07 | 0.50 |
| Male only | Model 1 | thalamus | 2.35 | 3.54 | 0.51 | 1.00 |
|  |  | caudate | -5.53 | 3.46 | 0.11 | 0.66 |
|  |  | putamen | -4.00 | 4.24 | 0.35 | 1.00 |
|  |  | pallidum | -2.31 | 1.30 | 0.08 | 0.53 |
|  |  | amygdala | 0.10 | 1.25 | 0.94 | 1.00 |
|  |  | hippocampus | -1.58 | 2.51 | 0.53 | 1.00 |
|  |  | NAcc | -0.78 | 0.60 | 0.19 | 0.97 |
|  |  | thalamus | -5.55 | 4.25 | 0.19 | 1.00 |
| Female only | Model 2 | caudate | 0.59 | 4.16 | 0.89 | 1.00 |
|  |  | putamen | -5.34 | 5.12 | 0.30 | 1.00 |
|  |  | pallidum | -1.24 | 1.57 | 0.43 | 1.00 |
|  |  | amygdala | 0.82 | 1.44 | 0.57 | 1.00 |
|  |  | hippocampus | 0.56 | 3.08 | 0.86 | 1.00 |
|  |  | NAcc | -1.55 | 0.77 | 0.04 | 0.30 |
| Male only | Model 2 | thalamus | 3.16 | 3.60 | 0.38 | 1.00 |
|  |  | caudate | -5.99 | 3.53 | 0.09 | 0.63 |
|  |  | putamen | -3.47 | 4.31 | 0.42 | 1.00 |
|  |  | pallidum | -2.16 | 1.33 | 0.11 | 0.63 |
|  |  | amygdala | 0.29 | 1.28 | 0.82 | 1.00 |
|  |  | hippocampus | -1.62 | 2.56 | 0.53 | 1.00 |
|  |  | NAcc | -0.84 | 0.61 | 0.17 | 0.85 |
| Female only | Model 3 | thalamus | -4.39 | 4.78 | 0.36 | 1.00 |
|  |  | caudate | -1.70 | 4.69 | 0.72 | 1.00 |
|  |  | putamen | -5.54 | 5.76 | 0.34 | 1.00 |
|  |  | pallidum | -1.35 | 1.77 | 0.45 | 1.00 |
|  |  | amygdala | 2.39 | 1.62 | 0.14 | 0.98 |
|  |  | hippocampus | 3.83 | 3.47 | 0.27 | 1.00 |
|  |  | NAcc | -1.14 | 0.86 | 0.18 | 1.00 |
| Male only | Model 3 | thalamus | 5.98 | 4.06 | 0.14 | 0.98 |
|  |  | caudate | -4.84 | 3.98 | 0.22 | 1.00 |
|  |  | putamen | -2.96 | 4.86 | 0.54 | 1.00 |
|  |  | pallidum | -1.52 | 1.50 | 0.31 | 1.00 |
|  |  | amygdala | 1.14 | 1.44 | 0.43 | 1.00 |
|  |  | hippocampus | 1.74 | 2.88 | 0.55 | 1.00 |
|  |  | NAcc | -0.24 | 0.69 | 0.72 | 1.00 |

*Table S5: Results from regression modelling of the association between the SRS and subcortical ROIs stratifying on sex. Corrected p-values were generated using holm correction for multiple testing. NAcc: nucleus accumbens. Model 1 was adjusted for child age, sex, ethnicity, family income, ICV, and ABCD recruitment site. Model 2 was adjusted for the covariates included in model 1 with the addition of cognition score. Model 3 was adjusted for the covariates included in model 2 with the addition of externalising symptoms and internalising symptoms.*

|  |  | **SRS** | | | |  | **SRS x sex** | | | |
| --- | --- | --- | --- | --- | --- | --- | --- | --- | --- | --- |
|  | **ROI** | **B** | **SE** | **p-value** | **holm p-value** |  | **B** | **SE** | **p-value** | **holm p-value** |
| Model 1 | thalamus | -7.82 | 4.46 | 0.08 | 0.48 |  | 10.68 | 5.55 | 0.05 | 0.38 |
|  | caudate | -0.65 | 4.36 | 0.88 | 1.00 |  | -4.23 | 5.43 | 0.44 | 1.00 |
|  | putamen | -6.00 | 5.35 | 0.26 | 1.00 |  | 1.64 | 6.67 | 0.81 | 1.00 |
|  | pallidum | -1.20 | 1.64 | 0.47 | 1.00 |  | -1.03 | 2.05 | 0.62 | 1.00 |
|  | amygdala | 0.94 | 1.55 | 0.54 | 1.00 |  | -1.06 | 1.93 | 0.58 | 1.00 |
|  | hippocampus | -0.39 | 3.19 | 0.90 | 1.00 |  | -1.38 | 3.97 | 0.73 | 1.00 |
|  | NAcc | -1.51 | 0.78 | 0.05 | 0.36 |  | 0.79 | 0.97 | 0.41 | 1.00 |
| Model 2 | thalamus | -5.85 | 4.49 | 0.19 | 1.00 |  | -5.85 | 4.49 | 0.19 | 1.00 |
|  | caudate | -0.08 | 4.40 | 0.99 | 1.00 |  | -5.05 | 5.49 | 0.36 | 1.00 |
|  | putamen | -4.86 | 5.38 | 0.37 | 1.00 |  | 0.97 | 6.72 | 0.89 | 1.00 |
|  | pallidum | -1.39 | 1.66 | 0.40 | 1.00 |  | -0.65 | 2.07 | 0.76 | 1.00 |
|  | amygdala | 0.93 | 1.56 | 0.55 | 1.00 |  | -0.99 | 1.95 | 0.61 | 1.00 |
|  | hippocampus | 0.52 | 3.21 | 0.87 | 1.00 |  | -2.39 | 4.01 | 0.55 | 1.00 |
|  | NAcc | -1.61 | 0.78 | 0.04 | 0.28 |  | 0.77 | 0.97 | 0.43 | 1.00 |
| Model 3 | thalamus | -3.48 | 4.75 | 0.46 | 1.00 |  | 9.02 | 5.60 | 0.11 | 0.75 |
|  | caudate | -0.55 | 4.65 | 0.91 | 1.00 |  | -4.95 | 5.49 | 0.37 | 1.00 |
|  | putamen | -4.58 | 5.70 | 0.42 | 1.00 |  | 0.85 | 6.72 | 0.90 | 1.00 |
|  | pallidum | -1.08 | 1.76 | 0.54 | 1.00 |  | -0.71 | 2.07 | 0.73 | 1.00 |
|  | amygdala | 2.10 | 1.65 | 0.20 | 1.00 |  | -1.19 | 1.95 | 0.54 | 1.00 |
|  | hippocampus | 4.21 | 3.39 | 0.21 | 1.00 |  | -3.07 | 4.00 | 0.44 | 1.00 |
|  | NAcc | -1.05 | 0.83 | 0.20 | 1.00 |  | 0.69 | 0.97 | 0.48 | 1.00 |

*Table S6: Results from regression modelling of the association between the SRS and subcortical ROIs with the inclusion of a sex X SRS interaction term. Corrected p-values were generated using holm correction for multiple testing. NAcc: nucleus accumbens. Model 1 was adjusted for child age, sex, ethnicity, family income, ICV, and ABCD recruitment site. Model 2 was adjusted for the covariates included in model 1 with the addition of cognition score. Model 3 was adjusted for the covariates included in model 2 with the addition of externalising symptoms and internalising symptoms.*
